# Nomophobia and Its Predictors: The Role of Psychological, Sociodemographic, and Internet Use Factors

**DOI:** 10.3390/ijerph22101495

**Published:** 2025-09-27

**Authors:** Inês Saraiva Ferreira, Belén Rando, António Esteves, Milena Castro, Inês Xavier, Ana Maria Abreu

**Affiliations:** 1 Health Sciences Faculty, Universidade Europeia, 1500-210 Lisboa, Portugal; antonioesteves@gmail.com (A.E.); milenaecastro@gmail.com (M.C.); ines.xavier@universidadeeuropeia.pt (I.X.); 2 Faculdade de Psicologia, Universidade de Lisboa, 1649-013 Lisboa, Portugal; 3 Center for Research in Neuropsychology and Cognitive and Behavioral Intervention (CINEICC), PsyAssessmentLab, Universidade de Coimbra, 3001-401 Coimbra, Portugal; 4 Centre for Public Administration and Public Policies (CAPP), Institute of Social and Political Sciences, Universidade de Lisboa, 1300-663 Lisboa, Portugal; mcalvo@iscsp.ulisboa.pt; 5 Insight, Piaget Research Center for Ecological Human Development, 2805-059 Almada, Portugal; anamariablom@gmail.com; 6 Center for Interdisciplinary Research in Health (CIIS), Universidade Católica Portuguesa, 1500-210 Lisboa, Portugal

**Keywords:** nomophobia, phubbing, sociodemographic factors, psychological variables, linear regression analysis

## Abstract

Nomophobia, or the fear of not being able to use a smartphone and/or the services, has gained increasing attention due to its growing prevalence. This study aimed to examine the prevalence of nomophobia and of potential variables associated with the phenomenon. Additionally, it sought to determine if the average of total nomophobia and the four second-order factors differed across gender. Finally, it analyzed the associations between nomophobia (overall and second-order factors) and psychological variables (self-esteem, loneliness, life satisfaction, and phubbing behavior), internet use, and sociodemographic characteristics. A cross-sectional survey was conducted with 306 participants (68.6% women), aged between 18 and 79 years (*M* = 38.0, *SD* = 16.3), using an online questionnaire. Descriptive statistics, independent samples *t*-tests comparing groups by gender, and bivariate correlations were computed. After, multiple linear regression analyses were performed to obtain parsimonious models with the most relevant variables (psychological variables, internet use, and sociodemographic characteristics) associated with overall nomophobia and its four dimensions. The results were generally consistent with the previous literature. Notably, gender and phubbing behavior were significantly associated with nomophobia. These findings contribute to a better understanding of the nomophobia phenomenon and may inform future interventions aimed at mitigating its potential impact on well-being.

## 1. Introduction

Technological advances and the digitalization of the media in recent decades have made the use of mobile phones and smartphones inseparable from our lives. Smartphones have become indispensable in the activities of daily life, serving a wide range of functions such as facilitating social interactions, payments and purchases, games, navigation systems, and access to countless information through applications and the Internet [1,2]. Some individuals even refer to these devices as extensions of their own body or an integral part of their identity [3]. However, the extensive use of these devices is not without risks, as excessive use can trigger negative emotional reactions and interfere with behavior and daily functioning [4].

The term *nomophobia* (short for “no-mobile-phone phobia”) was first introduced in 2008 in a study by YouGov (UK), commissioned by the UK Post Office [5]. It describes a situational phobia characterized by the fear of being unable to use a smartphone and/or access its services [6]. In the context of this study, the construct encompasses explicitly the fear of being unable to communicate or access information, losing connectedness, and giving up the convenience that smartphones provide [7]. Although it is not formally recognized as a clinical entity [8], existing literature suggests a positive relationship between nomophobia and emotional symptoms, namely depression, anxiety, phobic, and obsession symptoms [1,9,10,11,12], as well as problematic use [13,14] and smartphone dependence [15,16]. These emotional and behavioral changes have been observed in individuals deprived of access to their smartphones.

Also associated with smartphone use, an emerging behavior known as *phubbing* has been identified. This term was formed from the combination of the words ‘phone’ and ‘snubbing’, referring to the act of ignoring individuals in face-to-face interactions, focusing on the smartphone itself [17]. To assess this construct, Karadağ et al. [18] developed the Phubbing Scale, which considers two dimensions of phubbing: communication disturbance and phone obsession. Later, Niu et al. [19] suggested that this behavior can lead to disconnection with others and a loss of interpersonal communication, increasing smartphone use, and potentially nomophobia. Although studies analyzing the association between nomophobia and phubbing are scarce, some research conducted with university students has specifically documented this association [20,21].

Examining other psychological variables, Ozdemir et al. [22] found that university students with higher levels of nomophobia tended to experience greater loneliness and lower self-esteem. Likewise, Lekra [23] identified a significant positive association between loneliness and nomophobia in adults. An increased sense of loneliness within family relationships, particularly among university students, has also been shown to heighten the risk of nomophobia [24].

Concerning self-esteem, Vagka et al. [25] reported a strong association, noting that students with low self-esteem were twice as likely to suffer from nomophobia compared to those with high self-esteem. This negative relationship between self-esteem and nomophobia has been supported by other studies as well [26,27]. These findings suggest that individuals with low self-esteem may be more vulnerable to problematic smartphone use. Smartphones can facilitate interaction and compensate for social needs, yet they may also distance individuals from real-world relationships and contribute to feelings of loneliness [28].

The extant literature presents inconclusive results regarding the relationship between life satisfaction and nomophobia. While several studies have reported a negative association between the two constructs [29], other findings indicate that this relationship is not statistically significant [30,31,32]. Perhaps, sample characteristics may explain these different results. To gain a more nuanced understanding of this relationship, new evidence is needed.

According to the literature review, we noted that the relationship between nomophobia and the mentioned psychological variables has been relatively understudied. Regarding sociodemographic variables, research conducted on adults from the general population across diverse age groups has indicated that women and younger individuals tend to exhibit higher levels of nomophobia [33,34,35]. In a study conducted by Galhardo et al. [36] involving Portuguese participants aged 18 to 59, women scored higher than men on the total nomophobia score and on three of its dimensions, which is consistent with previous research [37,38]. However, no significant differences were observed between men and women participants in relation to the “Losing connectedness” dimension of nomophobia. This finding suggests that, despite differences in other factors, both men and women appear to experience comparable sentiments of a lack of omnipresence and connectivity, as well as a sense of disconnection from their online identities, particularly within the context of social media and online networks. Also, in the study by Galhardo et al. [36], a significant negative association was found with nomophobia and years of schooling, suggesting that an increased level of education is associated with a reduced risk of anxiety when smartphone use is not possible.

Finally, studies have documented a significant positive relationship between daily internet usage time and levels of nomophobia [25,39], thus constituting a potentially relevant factor in the analysis of this phenomenon.

In line with this literature, the present study aimed to expand the knowledge on nomophobia, considering variables potentially associated with this phenomenon. To this end, the prevalence of nomophobia, self-esteem, loneliness, life satisfaction, phubbing, and time spent on the internet was examined. Additionally, this study aimed to determine if the average score on nomophobia (overall and the four second-order factors) differed across genders, as a previous study involving Portuguese participants had shown that women scored higher than men on the overall nomophobia and on three of its dimensions. So, we sought to determine whether our results would corroborate previous findings. Finally, relationships between nomophobia, psychological variables, internet use, and sociodemographic characteristics were analyzed to obtain parsimonious models with the main variables associated with the phenomenon. As a specific objective, the present work aimed to know if variables related to the four second-order factors of nomophobia and to overall nomophobia were similar or distinct.

Therefore, this study is justified by the need to further explore nomophobia in a non-university adult population, which remains understudied in the literature, particularly considering the combined role of psychological, sociodemographic, and internet use variables. In a post-pandemic context, marked by increased dependence on digital technologies and the growing integration of smartphones into daily life, examining the factors associated with this phenomenon becomes particularly relevant. The results of this study are expected to contribute not only to the advancement of scientific knowledge but also to the development of strategies that promote mental health, increase digital literacy, and inform the development of preventive interventions aimed at mitigating the potential negative impact of technological dependence on individual well-being.

## 2. Methods

### 2.1. Participants

Participants were recruited based on the following inclusion criteria: individuals aged 18 or older, residents in Portugal, having written comprehension of the Portuguese language, and smartphone users. The sample consisted of 306 participants, 210 women and 96 men, aged between 18 and 79 (*M* = 38.0, *SD* = 16.3). Further sociodemographic characteristics of the sample are presented in Table A1.

### 2.2. Measures

The assessment protocol included sociodemographic data and information regarding time spent on the internet, as well as five questionnaires designed to evaluate the psychological constructs (nomophobia, self-esteem, perceived loneliness, life satisfaction, phubbing behavior), as described below.

The Nomophobia Questionnaire (NMP-Q) [7] consists of 20 items that inform of fear of being without access to a mobile phone (overall nomophobia), as well as of four second-order factors: Factor 1, not being able to communicate (6 items), that is, feelings of failing to communicate and being accessible for others; Factor 2, losing connectedness (5 items), that reflects feelings of missing continuous presence and being detached from online social media; Factor 3, not being able to access information (4 items), as discomfort of not accessing to a widespread information; and Factor 4, unwillingness to give up smartphone convenience (5 items), related to feelings of convenience of having a smartphone and the desire to own it. Responses are recorded on a 7-point Likert-type scale, where 1 = totally disagree and 7 = totally agree. The total score can range from 20 to 140 points, with higher scores indicating greater levels of nomophobia. According to the authors, the NMP-Q total score should be interpreted as follows: 20 = absence of nomophobia; 21 to 59 = mild level of nomophobia; 60 to 99 = moderate level of nomophobia; 100 to 140 = severe nomophobia. The Cronbach’s alpha coefficient for the total score was 0.96 in the Portuguese adaptation study [36], hereafter called the NMP-Q-PT. Using this adapted version in the present work, we obtained the same Cronbach’s alpha coefficient.

The refined version [40] of the Phubbing Scale (PPS) [18] consists of eight items (instead of 10) divided into two dimensions: Communication Disturbance (items 1 to 4), which assess the frequency an individual disturbs face-to-face communication due to smartphone use (e.g., “When I’m with my friends, I often get distracted by my phone”), and Phone Obsession (items 5 to 8), which measures the need to use a smartphone in non-face-to-face settings (e.g., “The first thing I do when I wake up is check my messages on my phone”). Responses are recorded on a 5-point Likert-type scale, ranging from 1 (never) to 5 (always). The total score ranges from 8 to 40. Higher scores reflect mobile phone addiction. In the Portuguese adaptation (hereafter called PPS-PT) [41], McDonald’s omega coefficient was 0.85 for communication disturbance and 0.76 for phone obsession. In the present study, Cronbach’s alpha reliability coefficient for the total score was 0.80.

The Single-Item Life Satisfaction Measure (SI-LS) assesses life satisfaction through the question [42] “On a scale from 1 to 10, how satisfied are you with your life?” Participants respond on a 1 to 10 points scale, where 1 = not at all satisfied and 10 = completely satisfied. Higher points on the scale indicate life satisfaction. Previous studies with the Portuguese version (hereafter called SI-LS-PT) support the psychometric adequacy of the factor structure and the reliability of this measure [43].

The Rosenberg Self-Esteem Scale (RSES) [44] is a self-assessment measure of global self-esteem, consisting of 10 items, 5 with a positive orientation and 5 with a negative orientation. It uses a 4-point Likert-type scale for responses, ranging from 1 = strongly agree to 4 = strongly disagree. The total score ranges from 10 to 40, with higher scores reflecting a greater level of self-esteem. The Portuguese adaptation of RSES [45] (hereafter called RSES-PT) reported a Cronbach’s alpha of 0.86. We used this adapted version in the current study and the Cronbach’s alpha was 0.89.

Version 3 of UCLA Loneliness Scale (UCLA-LSV3) [46] comprehends 20 items that assess both subjective feelings of loneliness and social isolation. It is a commonly used measure in studies on stress, anxiety, depression, and physical health impacts. Responses are provided on a 4-point Likert-type scale, ranging from 1 = “never” to 4 = “always.” The total score ranges from 20 to 80. Higher scores mean higher levels of perceived loneliness. In the Portuguese adaptation of the scale (hereafter called UCLA-LSV3-PT) [47], the Cronbach’s alpha coefficient for the total score was 0.91. We used UCLA-LSV3-PT in the present research, and the Cronbach’s alpha was 0.92.

### 2.3. Procedures

The Ethics Committee of the Universidade Europeia (Lisbon) approved this study. In accordance with the Declaration of Helsinki of the World Medical Association and the Code of Ethics of the American Psychological Association, participation was voluntary, and all data were anonymized in compliance with European data privacy and security regulations [General Data Protection Regulation (EU) 2016/679 of the European Parliament and of the Council of 27 April 2016].

The instruments were administered through an online questionnaire disseminated via email and social networks, using a non-probability snowball sampling method that relied on the researchers’ personal and professional networks. Before accessing the questionnaire, participants were presented with an informed consent form outlining the study objectives, participation conditions, inclusion criteria, data management procedures, and the identities of the researchers. Only those who provided consent were able to proceed with the survey. On average, completion of the questionnaire required approximately 10–15 min. To ensure data quality, responses with unusually short completion times (less than five minutes) were excluded from the analyses, as these were deemed incompatible with attentive completion of the protocol. Data were collected between 1 and 31 January 2023, and no incentives were offered for participation.

### 2.4. Data Analysis

Analyses were performed using IBM SPSS Statistics 30.0. A descriptive analysis was conducted on nomophobia (NMP-Q-PT—Total and the four second-order factors), other psychological variables, sociodemographic factors, and time spent on the internet during weekdays and weekends. In addition, independent samples *t*-tests were performed to determine if the average scores for overall nomophobia and the four dimensions differed across genders. To this purpose, we first examined the normality of the dependent variables using the Kolmogorov–Smirnov (K-S) test and the homogeneity of variance through Levene’s test. In general, the data were not normally distributed. Nevertheless, the *t*-test is quite robust, particularly with large samples and the absence of outliers [48]. Later, bivariate correlations between nomophobia (overall and its dimensions) and the continuous variables were calculated using Pearson’s correlation coefficient. Finally, a series of multiple linear regression analyses were conducted to identify the best set of variables that account for the variability of nomophobia. In regression analyses, nomophobia and the four dimensions were the response variables. The independent variables were the following: gender (man, woman); age (years); marital status (single, married, separated, widowed); occupation (student, working student, employed, unemployed, retired); educational level (9th grade, secondary/technological/vocational, bachelor’s degree, master’s degree, Ph.D. degree); phubbing behavior from PPS-PT (total score); self-esteem from RSES-PT (total score); perceived loneliness from UCLA-LSV3-PT (total score); life satisfaction from SI-LS-PT (total score); internet use on weekdays (up to 1 h., 1–2 h, 3–4 h, 5–6 h, 7–8 h, 8+ h.); and, internet use on weekend days (up to 1 h., 1–2 h, 3–4 h, 5–6 h, 7–8 h, 8+ h.). Categorical variables were transformed into dummy variables, with one dummy variable serving as reference. The reference dummy variable in each case was: gender (man), marital status (single), occupation (student), and education level (secondary/professional or other). Stepwise method was used. Concerning the assumptions of multiple linear regressions, linearity, absence of redundance, and independence, normality and homogeneity of residuals variance were examined. To this end, the following procedures were undertaken: (a) assessment of residual normality through the normal probability plot; (b) evaluation of residual independence and homogeneity using the scatterplot of standardized residuals and standardized predicted values (in case of suspected heteroscedasticity, White’s test was applied); (c) verification of the absence of autocorrelation in the residuals using the Durbin-Watson statistic; (d) examination of the Variance Inflation Factor (VIF < 10) and the Condition Index (CI < 30) to confirm the absence of multicollinearity T [49,50].

## 3. Results

### 3.1. Sociodemographic Characteristics of the Sample

Table A1 presents the sociodemographic characteristics of the 306 participants. The majority of the sample were women (68.6%), and participants ranged in age from 18 to 79 years, with the largest groups being 26–49 years (36.3%) and 18–25 years (35.6%). Nearly half of the participants reported having a bachelor’s degree (48.7%), while 31.0% had completed secondary/technological/vocational education, and smaller proportions reported holding a master’s (13.1%) or doctoral degree (4.2%). Regarding occupational status, most participants were employed (55.9%), followed by students (22.2%) and working students (11.8%), with smaller percentages reporting retirement (7.5%) or unemployment (2.6%). In terms of marital status, approximately half were single (51.0%), 37.6% were married, 10.1% were separated/divorced, and 1.3% were widowed.

### 3.2. Prevalence of Nomophobia, Psychological Variables and Time Spent on the Internet

Table A2 and Table A3 show that, on average, participants in this study expressed a moderate fear of being without access to their mobile phones [7]. Since 75% of participants revealed scores on overall nomophobia less than 99, it can be said that most of the sample showed mild to moderate levels of nomophobia. Examining the four dimensions, half of the respondents obtained scores that can be considered close to a mild level of fears of losing connectedness (NMP-Q-PT—F2) and not being able to access to information (NMP-Q-PT—F3), but close to moderate level of fears of not being able to communicate (NMP-Q-PT—F1) and giving up convenience of smartphone (NMP-Q-PT—F4). Comparing by gender, women showed higher average scores on overall and the four second-order factors of nomophobia than men. *t* tests revealed statistically significant differences between women and men in total score of nomophobia and in NMP-Q-PT—F1 and NMP-Q-PT—F4. Considering the severity levels of nomophobia by gender, the results revealed higher mean scores and slightly lower standard deviations for women at the mild and moderate levels. At the severe level, women and men presented very similar mean scores and standard deviations (Table A4) [36].

In relation to PPS-PT, the sample seemed to be characterized by a low level of phubbing, since scores ranged up to 20 points for 50% of participants and up to 24 points for 75% of the sample. Concerning the other psychological variables, the participants tended to be satisfied with life (as measured by the SI-LS-PT), exhibited moderate to high levels of self-esteem, and had a low perception of loneliness.

Regarding the time spent on the internet on weekdays, a small number of participants (2.9%) reported using the internet for up to an hour, while 17.3% spent between one and two hours online. A quarter of respondents (24.8%) reported spending three to four hours online, while 22.2% reported spending five to six hours. Additionally, 11.8% of participants reported using the internet for seven to eight hours, and 20.9% were aware of exceeding eight hours on weekdays. Regarding internet usage habits on weekends, a small percentage of respondents (6.2%) reported using the internet for up to an hour, while 21.3% indicated spending one to two hours online, and a higher percentage of participants (29.1%) stated spending three to four hours online. Moreover, 16.7% admitted to spending five to six hours on the internet on weekends, 14.1% acknowledged using the internet for five to eight hours, and 12.7% reported exceeding eight hours online on weekends. Therefore, the time spent on the internet on weekdays and weekend days was relatively varied, but respondents tended to be moderate in the time they spent online.

### 3.3. Bivariate Correlations

Table A5 presents Pearson correlation coefficients between nomophobia (first and the four second-order factors) and the other continuous measures. The strength of the relationships is interpreted according to the criteria outlined by Cohen et al. [50], who consider an association can be small (r = 0.10 to 0.29), moderate (r = 0.30 to 0.49), large (r = 0.50 to 0.69), very large (r = 0.70 to 0.89), nearly perfect (r ≥ 0.90), and perfect (r = 1).

Focusing on the relationship between nomophobia (first- and second-order factors) and other psychological variables, as well as time spent online on weekdays and weekend days, it is possible to observe significant negative but small associations between self-esteem and nomophobia (first and second-order factors). In addition, both variables that measure time spent on the internet are positively related to overall nomophobia and the four second-order factors, but again these associations tend to be weak. However, when considering phubbing behavior, we found stronger positive associations, some of which were nearly as high as those observed among the second-order factors of nomophobia. In contrast, there are no significant relationships regarding life satisfaction, and only a significant positive but small association exists between perceived loneliness and fear of giving up smartphone convenience (NMP-Q-PT—F4).

Concerning age, overall nomophobia and its dimensions tended to decrease as the age of the participants increased. These associations are small too, but significant. Thus, younger people scored higher in these types of fear.

### 3.4. Multiple Linear Regression Models

Before conducting regression analyses, assumptions were tested. Linearity, absence of redundance, independence, and normality of residuals were confirmed. However, the homogeneity of variance assumption was not met for the regression models of Factor 1 (not being able to communicate), Factor 2 (losing connectedness), and Factor 4 (giving up smartphone convenience), as indicated by White’s test (χ^2^(4) = 11.945, *p* = 0.018; χ^2^(2) = 16.714, *p* < 0.001; χ^2^(4) = 10.094, *p* = 0.039, respectively). Variable transformations did not improve the homogeneity of variance. Although heterogeneity of residuals is undesirable, it does not bias parameter estimation [49,50], so analyses proceeded with the original variables.

To find the best set of variables that account for the variability of overall nomophobia and for the second-order factors, multiple regression analyses were conducted. Although the dimensions of nomophobia showed very large associations to NMP-Q-PT—Total, we wanted to know if respective regression models would reflect any difference, since analyses would be performed using several sociodemographic variables (gender, age, marital status, occupation, and educational level), phubbing behavior, life satisfaction, self-esteem, perceived loneliness, internet use on weekdays, and internet use on weekend days. Results are in Table A6.

A globally significant model was obtained for NMP-Q-PT—Total, with two variables that explain 44.7% of the variability. Phubbing behavior is the most relevant variable in this model. According to results, the fear of being without access to a smartphone increases on average by 3.172 units for every unit increase in phubbing, holding the gender variable constant. In addition, the overall nomophobia is, on average, six units higher among women compared to men, when phubbing is held constant.

The regression model obtained for NMP-Q-PT—F1 (not being able to communicate) uses phubbing and gender as variables that explain 33.2% of the variation in this fear. Holding gender constant, the fear of not being able to communicate rises by an average of 1.023 units for each unit that phubbing increases. Additionally, this fear is, on average, four units higher in women than in men, when phubbing remains constant.

With respect to NMP-Q-PT—F2 (losing connectedness) and NMP-Q-PT—F3 (not being able to access information), the regression analyses yielded a model with phubbing behavior as a single variable accounting for 29.5% and 36% of the variance, respectively. Considering the results, the fear of losing connectedness increases, on average, 0.711 units for each unit that phubbing rises, and fear of not being able to access information increases, on average, 0.623 units.

Finally, the regression model obtained for NMP-Q-PT—F4 (giving up smartphone convenience) again uses phubbing behavior and gender as variables explaining 38.9% of the variability. Holding gender constant, each unit of increase in phubbing raises the fear of giving up the mobile phone convenience by an average of 0.816 units. Additionally, this fear is, on average, 1.691 units higher in women than in men, when phubbing remains constant.

## 4. Discussion

The present work aimed to improve the knowledge of nomophobia, considering variables potentially associated with this phenomenon, including psychological variables (self-esteem, loneliness, life satisfaction, and phubbing behavior), sociodemographic factors, and internet usage time. More specifically, it sought to examine the prevalence of nomophobia and its association with these variables. To this end, data were collected from a sample of adults from the general population residing in Portugal who were smartphone users. In terms of sociodemographic distribution, this sample is broader than previous studies, which predominantly included adolescents and university students [4,6].

In this study, participants showed, on average, a moderate level of total nomophobia, characterized by a mild level of fear of losing connectedness and not being able to access information, and a moderate level of fear of not being able to communicate and giving up the convenience of a smartphone. Our results are consistent with those found by Galhardo et al. [36] in an earlier research with undergraduates and the general population in Portugal [21,22,23,24,25,26,27,28,29,30,31,32,33] which reinforces the idea that nomophobia is a common phenomenon. In line with those authors, we also found that women exhibited higher levels of nomophobia, particularly at mild and moderate levels. However, we only obtained significant differences in overall nomophobia and in fears of not being able to communicate and giving up the smartphone convenience, whereas Galhardo et al. [36] also found significant differences in fear of not being able to access information. Although our sample had a higher mean age (*M* = 38.0, *SD* = 16.3 vs. *M* = 22.95, *SD* = 5.36) and was recruited through non-probabilistic sampling, it is important to note the similarity of findings. It is also possible that the COVID-19 pandemic may have reduced gender disparities regarding fear of not being able to access information. Concerning fear of losing connectedness, average scores were higher in our research and gender differences remained. A possible explanation is the growing importance of online connection in daily life. In a changing world where people increasingly rely on the internet to manage multiple tasks and where artificial intelligence is becoming a core tool, staying connected has become essential.

As previously mentioned, participants in the present study reported low levels of phubbing and perceived loneliness, high life satisfaction, and moderate-to-high self-esteem. This profile contrasts with samples of university students, who typically report higher phubbing and loneliness scores [14,20,21]. Prior research indicates that individuals with severe nomophobia typically present with higher phubbing and loneliness, lower life satisfaction, and reduced self-esteem [22,25,26,30,32]. Thus, our findings are consistent with the literature, as the majority of participants in this study exhibited only mild to moderate levels of nomophobia, which is not usually associated with such negative psychological outcomes [21,22,29].

Regarding the relationship between nomophobia and the mentioned constructs, it is particularly noteworthy that phubbing exhibits a strong positive association. This result is consistent with prior studies conducted with samples of university students [20,21], confirming that individuals who engage more in phubbing behaviors are also more likely to display higher nomophobia levels. Perhaps, this result may be attributed to a pervasive fear of disconnection, which drives individuals to engage with their smartphones continuously, even during face-to-face interactions. At the same time, smartphones may serve as tools for regulating negative emotions and as instruments of social control in interpersonal situations perceived as difficult or undesirable [20,21].

Although self-esteem and age showed a modest association with overall nomophobia and the four dimensions, these significative and inverse relationships with these types of fear are in line with preliminary investigation [22,25,26,27,33,34,35]. It is important to note that participants in the research tended towards homogeneity in self-esteem, as well as in life satisfaction, according to descriptive statistics, which could explain the observed lower relationship with nomophobia and may have influenced correlation values obtained between life satisfaction and nomophobia, but possibly not affecting significance, since values obtained for life satisfaction were very low. In this sense, our results support the absence of association between the cognitive component of subjective well-being and nomophobia as previously found by several authors [30,31,32]. On the other hand, our findings support the positive association between internet usage and nomophobia too [25,39,51]. Similar associations have been reported by Jeong et al. [16] and Samaha and Hawi [32], who showed that extended online activity predicts greater smartphone dependence and nomophobia. This seemed to be expected, as nowadays the internet is essential for communication, being accessible to others, staying present, and avoiding detachment from online social media. It is also a key source of information, and having a smartphone is a convenient option. In short, the internet enables nomophobic individuals to obtain what they feel they need.

In our study, participants reported wide variability in daily internet use, with a considerable proportion spending more than six hours online, particularly on weekends. Although higher internet use was positively but weakly associated with nomophobia, this finding is consistent with previous research indicating that excessive screen time can increase the risk of problematic smartphone behaviors [25,51]. Currently, there are no specific evidence-based recommendations regarding optimal internet use for adults. However, international guidelines on digital well-being [52] emphasize the importance of balanced use, highlighting that online activities should not interfere with sleep, physical activity, work, or social interactions. Thus, instead of setting a strict hourly threshold, recommendations tend to focus on promoting mindful use, setting personal boundaries, and monitoring the potential impact of online behaviors on daily functioning and well-being.

In contrast, loneliness only exhibited a modest positive significant relationship with relinquishing the convenience of the smartphone, while the prior literature reported a significant association with overall nomophobia, either in undergraduate students or adults [22,23]. A possible explanation is that our participants, being older and less lonely on average than typical university samples, displayed a weaker link between loneliness and global nomophobia [23,24]. Since the dimension of nomophobia linked to convenience reflects practical or habitual concerns, this result could explain why individuals who perceive themselves as lonely show a fear of giving up the convenience of having a smartphone. In any case, more evidence is needed to clarify these mixed findings. This reinforces the conceptual proposal of Karadağ et al. [18], who suggested that phubbing is a behavioral expression of problematic smartphone use and is closely intertwined with nomophobia.

According to the results of multiple linear regressions, phubbing and gender had greater explanatory power than other variables considered in the analyses. Our findings highlight phubbing as a central factor, as overall nomophobia and the four factors increased with higher phubbing levels, and are in line with the positive association observed in prior investigations [20,21].

Additionally, gender emerged as a predictor of overall nomophobia, as did the fear of not being able to communicate and giving up smartphone convenience. These results are in accordance with those obtained by comparing women and men, and also demonstrate the relevance of gender among other sociodemographic and psychological variables as a factor accounting for some variability in these types of fear. Moreover, these results corroborate the findings of Galhardo et al. [36] about women being more susceptible to overall nomophobia and three of the dimensions. Comparable outcomes have been observed in other cultural contexts [11,33], although the magnitude of the effect differs across countries. Bearing in mind the negative association between nomophobia and age in our work, probably this susceptibility occurs mainly in younger women. In that case, it would be consistent with previous research, which revealed that women and younger individuals tend to show higher levels of nomophobia [33,34,35].

This study sought to contribute to current knowledge of nomophobia and to a better understanding of a phenomenon with growing relevance due to its potential impact on psychological well-being and lifestyle across different stages of the life cycle. Recognizing the clinical and educational implications of nomophobia is crucial for raising awareness about its assessment and identification. Such knowledge can contribute to the development of interventions focused on target populations, including psychoeducational and preventive programs aimed at addressing and mitigating this issue.

In particular, understanding the relationship between nomophobia and phubbing is essential for designing educational initiatives that promote healthy technology use and strengthen social skills. These programs should focus on increasing awareness of the negative consequences associated with these behaviors while fostering practices that support full engagement in face-to-face social interactions.

Finally, it is important to recognize some constraints of this study, such as the use of the snowball strategy, which does not guarantee the representativeness of the sample. Additionally, self-report questionnaires may be subject to social desirability bias or misinterpretation, potentially influencing the results. Also, life satisfaction was assessed using the single item of Jovanovic [42]. Despite its practical utility, perhaps results may vary when using a different instrument or at least this possibility should be explored. Future research also may consider other psychological variables, such as anxiety and depression, and other well-being measures like human flourishing to explore their interrelation with nomophobic behavior.

## 5. Conclusions

This study contributes to a deeper understanding of nomophobia among adults, highlighting its associations with psychological, sociodemographic, and internet use factors. The findings reinforce the conceptualization of nomophobia as a multidimensional phenomenon with potential associations with psychological and everyday functioning. Phubbing emerged as the strongest predictor, underscoring the importance of addressing excessive smartphone use during interpersonal interactions through targeted educational and preventive efforts. Identifying vulnerable groups is particularly relevant for designing tailored psychoeducational programs that promote healthy technology use and foster stronger face-to-face social engagement.

## Data Availability

The data presented in this study are available on request from the corresponding author, I.S.F.

## Abbreviations

The following abbreviations are used in this manuscript:

NMP-Q	Nomophobia Questionnaire
PPS	Phubbing Scale
RSES	Rosenberg Self-Esteem Scale
SI-LS	Single-Item Life Satisfaction Measure
UCLA-LSV3	UCLA Loneliness Scale–version 3

## Appendix A

**Table A1:** The Sociodemographic Characteristics of the Study Sample (*n* = 306).

Variable	*n*	%
Gender		
Woman	210	68.6
Man	96	31.4
Age (years)		
18–25	109	35.6
26–49	111	36.3
50–79	86	28.1
Educational Level		
9th grade	9	2.9
Secondary/Technological/Vocational Education	95	31.0
Bachelor’s Degree	149	48.7
Master’s Degree	40	13.1
Ph.D. Degree	13	4.2
Occupation		
Student	68	22.2
Working Student	36	11.8
Employed	171	55.9
Unemployed	8	2.6
Retired	23	7.5
Marital Status		
Single	156	51
Married	115	37.6
Separated/Divorced	31	10.1
Widowed	4	1.3

**Table A2:** Descriptive Statistics of Overall Nomophobia, the Second-Order Factors of Nomophobia and Other Psychological Variables (*n* = 306).

	*Min.*	*Max.*	*M*	*SD*	*Q*1	*Q*2	*Q*3
NMP-Q-PT—Total	20	131	68.26	26.79	45.55	69.64	87.45
NMP-Q-PT—F1	6	42	24.17	10.53	14.86	25.00	32.76
NMP-Q-PT—F2	5	35	13.82	7.14	7.85	12.24	19.12
NMP-Q-PT—F3	4	28	15.15	5.87	10.59	15.21	19.32
NMP-Q-PT—F4	5	35	15.12	7.36	9.14	14.04	20.27
PPS-PT (Phubbing)	9	37	20.27	5.69	16.09	19.89	24.13
RSES-PT (Self-esteem)	15	40	31.44	5.19	28.15	31.48	35.44
(UCLA-LSV3-PT (Loneliness)	20	71	39.25	9.74	32.41	38.44	44.94
SI-LS-PT (Life satisfaction)	1	10	7.38	1.63	6.44	7.53	8.54

Note. Scores range: NMP-Q-PT—Total (fear of being without access to a mobile phone) = 20 to 140; NMP-Q-PT—F1 (not being able to communicate) = 6 to 42; NMP-Q-PT—F2 (losing connectedness) = 5 to 35; NMP-Q-PT—F3 (not being able to access information) = 4 to 28; NMP-Q-PT—F4 (giving up convenience) = 5 to 35; PPS_PT (Phubbing) = 8 to 40; RSES_PT (Self-esteem) = 10 to 40; UCLA-LSV3-PT (loneliness) = 20 to 80; SI-LS-PT (Life Satisfaction) = 1 to 10.

**Table A3:** Prevalence of Overall Nomophobia and Its Dimensions by Gender and Independent Samples *t*-Tests Comparing Groups by Gender (*n* = 306).

	Women (*n* = 210)		Men (*n* = 96)		*t*(304)	*Sig.*
	*M*	*SD*	*M*	*SD*		
NMP-Q-PT—Total	70.91	26.39	62.48	26.87	−2.58	0.010
NMP-Q-PT—F1	25.62	10.39	21.00	10.17	−3.63	<0.001
NMP-Q-PT—F2	13.92	7.10	13.61	7.25	−0.35	0.730
NMP-Q-PT—F3	15.50	5.74	14.40	6.10	−1.53	0.127
NMP-Q-PT—F4	15.87	7.38	13.47	7.08	−2.68	0.008

Note. Scores range: NMP-Q-PT—Total (fear of being without access to a mobile phone) = 20 to 140; NMP-Q-PT—F1 (not being able to communicate) = 6 to 42; NMP-Q-PT—F2 (losing connectedness) = 5 to 35; NMP-Q-PT—F3 (not being able to access information) = 4 to 28; NMP-Q-PT—F4 (giving up convenience) = 5 to 35.

**Table A4:** Prevalence of Nomophobia Severity Levels, Both Overall and by Gender.

	Overall (*n* = 306)		Women (*n* = 210)		Men (*n* = 96)	
	*M*	*SD*	*M*	*SD*	*M*	*SD*
Mild	42.73	10.10	42.55	9.36	40.89	10.16
Moderate	80.64	10.48	81.29	10.02	78.97	11.57
Severe	112.64	8.81	112.53	8.92	113.00	8.97

Note. Scores range: Mild = 21 to 59; Moderate = 60 to 99; Severe = 100 to 140.

**Table A5:** Pearson Correlation Coefficients (*n*= 306).

Variable	1	2	3	4	5	6	7	8	9	10	11	12
1. NMP-Q-PT—Total	—											
2. NMP-Q-PT—F1	0.896 **	—										
3. NMP-Q-PT—F2	0.847 **	0.646 **	—									
4. NMP-Q-PT—F3	0.814 **	0.619 **	0.622 **	—								
5. NMP-Q-PT—F4	0.888 **	0.711 **	0.694 **	0.679 **	—							
6. PPS-PT (Phubbing)	0.646 **	0.526 **	0.543 **	0.613 **	0.584 **	—						
7. SI-LS-PT (Life satisfaction)	−0.055	−0.045	−0.011	−0.026	−0.104	−0.033	—					
8. RSES-PT (Self-esteem)	−0.191 **	−0.121 *	−0.160 **	−0.165 **	−0.236 **	−0.225 **	0.556 **	—				
9. UCLA-LSV3-PT (Loneliness)	0.092	0.057	0.063	0.039	0.161 **	0.058	−0.571 **	−0.557 **	—			
10. Internet use on weekdays	0.208 **	0.172 **	0.182 **	0.227 **	0.153 **	0.353 **	−0.019	−0.211 **	0.108	—		
11. Internet use on weekends	0.195 **	0.103	0.198 **	0.224 **	0.191 **	0.293 **	−0.198 **	−0.360 **	0.200 **	0.597 **	—	
12. Age	−0.221 **	−0.176 **	−0.151 **	−0.264 **	−0.197 **	−0.262 **	0.122 *	0.310 **	−0.032	−0.291 **	−0.427 **	—

Note. NMP-Q-PT-Total (fear of being without access to a mobile phone); NMP-Q-PT—F1 (not being able to communicate); NMP-Q-PT—F2 (losing connectedness); NMP-Q-PT—F3 (not being able to access information); NMP-Q-PT—F4 (giving up convenience). * *p* < 0.005, ** *p* < 0.001.

**Table A6:** Adjusted R2, Global ANOVA and Coefficients of Regression Models.

Model	*B*	*SE*	*β*	*t*	*Sig.*
NMP-Q-PT—Total Adj. R^2^ = 0.447 F (2, 236) = 97.230, *p* < 0.001 (Constant)	2.286	5.345		0.054	0.957
Phubbing	3.172	0.229	0.667	13.827	<0.001
Gender (woman)	5.919	2.817	0.101	2.101	0.037
NMP-Q-PT—F1 Adj. R^2^ = 0.332 F (2, 236) = 60.091, *p* < 0.001 (Constant)	0.834	2.265		0.368	0.713
Phubbing	1.023	0.097	0.557	10.516	<0.001
Gender (woman)	3.960	1.194	0.176	3.317	0.001
NMP-Q-PT—F2 Adj. R^2^ = 0.295 F (1, 237) = 100.539, *p* < 0.001 (Constant)	−0.598	1.518		−0.394	0.694
Phubbing	0.711	0.071	0.546	10.027	<0.001
NMP-Q-PT—F3 Adj. R^2^ = 0.360 F (1, 237) = 134.745, *p* < 0.001 (Constant)	2.774	1.149		2.414	0.017
Phubbing	0.623	0.054	0.602	11.608	<0.001
NMP-Q-PT—F4 Adj. R^2^ = 0.389 F (2, 236) = 76.738, *p* < 0.001 (Constant)	−2.524	1.551		−1.627	0.105
Phubbing	0.816	0.067	0.621	12.255	<0.001
Gender (woman)	1.691	0.818	0.105	2.069	0.040

Note. NMP-Q-PT—Total (fear of being without access to a mobile phone); NMP-Q-PT—F1 (not being able to communicate); NMP-Q-PT—F2 (losing connectedness); NMP-Q-PT—F3 (not being able to access information); NMP-Q-PT—F4 (giving up convenience).
